# Targeted Disruption of the *Inhibitor of DNA Binding 4* (*Id4*) Gene Alters Photic Entrainment of the Circadian Clock

**DOI:** 10.3390/ijms22179632

**Published:** 2021-09-06

**Authors:** Giles E. Duffield, Maricela Robles-Murguia, Tim Y. Hou, Kathleen A. McDonald

**Affiliations:** 1 Department of Biological Sciences, Galvin Life Science Center, University of Notre Dame, Notre Dame, IN 46556, USA; roblesmm@gmail.com (M.R.-M.); tim.yt.hou@gmail.com (T.Y.H.); kathleen.mcdonald.13@gmail.com (K.A.M.); 2 Eck Institute for Global Health, University of Notre Dame, Notre Dame, IN 46556, USA

**Keywords:** photoentrainment, circadian rhythm, phase shift, light, circadian clock, pupillometry, suprachiasmatic nucleus, Inhibitor of DNA binding, ID4, transcriptional inhibitor

## Abstract

*Inhibitor of DNA binding* (*Id*) genes comprise a family of four helix–loop–helix (HLH) transcriptional inhibitors. Our earlier studies revealed a role for ID2 within the circadian system, contributing to input, output, and core clock function through its interaction with CLOCK and BMAL1. Here, we explore the contribution of ID4 to the circadian system using a targeted disruption of the *Id4* gene. Attributes of the circadian clock were assessed by monitoring the locomotor activity of *Id4*−/− mice, and they revealed disturbances in its operation. *Id4*-mutant mice expressed a shorter circadian period length, attenuated phase shifts in responses to continuous and discrete photic cues, and an advanced phase angle of entrainment under a 12:12 light:dark cycle and under short and long photoperiods. To understand the basis for these properties, suprachiasmatic nucleus (SCN) and retinal structures were examined. Anatomical analysis reveals a smaller *Id4*−/− SCN in the width dimension, which is a finding consistent with its smaller brain. As a result of this feature, anterograde tracing in *Id4*−/− mice revealed retinal afferents innovate a disproportionally larger SCN area. The *Id4*−/− photic entrainment responses are unlikely to be due to an impaired function of the retinal pathways since *Id4*−/− retinal anatomy and function tested by pupillometry were similar to wild-type mice. Furthermore, these circadian characteristics are opposite to those exhibited by the *Id2*−/− mouse, suggesting an opposing influence of the ID4 protein within the circadian system; or, the absence of ID4 results in changes in the expression or activity of other members of the *Id* gene family. Expression analysis of the *Id* genes within the *Id4*−/− SCN revealed a time-of-day specific elevated *Id1*. It is plausible that the increased *Id1* and/or absence of ID4 result in changes in interactions with bHLH canonical clock components or with targets upstream and/or downstream of the clock, thereby resulting in abnormal properties of the circadian clock and its entrainment.

## 1. Introduction

Many aspects of biochemistry, physiology, and behavior are organized around a 24-h rhythm, which is driven by an endogenous circadian clock [1,2]. Circadian organization in single cells is based on a series of interlocked autoregulatory molecular transcriptional-translational feedback loops (TTFLs) comprised of ‘clock genes’. The basic helix–loop–helix (bHLH)/Per-ARNT-SIM (PAS) transcription factors CLOCK, NPAS2, and BMAL1 contribute a positive loop, *period* (*per1*, *per2*) and *cryptochrome* (*cry1*, *cry2*) genes contribute a negative loop, and the nuclear receptors REVERB and ROR provide an interlocking loop [1,2]. In mammals, the master circadian oscillator resides within the hypothalamic suprachiasmatic nucleus (SCN), which regulates the rhythmic physiology and behavior and coordinates peripheral clocks throughout the body [1,2,3,4,5]. Disturbances to the circadian system have been linked to the development of metabolic disease including diabetes and obesity, cardiovascular disease, tumorigenesis, sleep–wake disorders, and mental illness [1,2].

Inhibitor of DNA binding (ID) proteins (ID1, ID2, ID3, and ID4) comprise a family of four helix–loop–helix (HLH) transcriptional inhibitors, and they regulate development and tumorigenesis [6]. They function at the molecular level as dominant negative transcriptional regulators of specific bHLH transcription factors. Their role in regulating adult physiology and behavior has not been extensively studied. Our previous investigations have revealed important roles for ID2 in the circadian system. *Id2* mRNA and protein are rhythmically expressed where found, including SCN, liver, and immortalized fibroblasts [7,8,9,10,11,12]. ID2 interacts with canonical clock proteins CLOCK and BMAL1 through their HLH domain, resulting in the inhibition of their transactivation potential by sequestering CLOCK and BMAL1 to the cytoplasm [7,13]. The expression profile of rhythmically expressed clock-controlled genes (CCGs) in the liver of *Id2*−/− mice is altered, revealing a role for ID2 in regulating the circadian clock *output pathways* [9]. *Id2*−/− mice express altered circadian locomotor activity and feeding behavior profiles [14,15,16]. Finally, ID2 contributes to the photoentrainment mechanism [7,17], in which *Id2*−/− mice are hyper-responsive to photic cues and can rapidly re-entrain to a new light/dark (LD) cycle in half the time as wild-type counterparts. Mice lacking *Id2* exhibit abnormally rapid entrainment in response to a large change in the photoschedule, corresponding to an increased magnitude of light-induced phase delays and a delayed phase angle of entrainment [7,17]. These phase-shifting responses are correlated with increased *per1* clock gene expression in both SCN and in cell lines derived from *Id2*−/− mice that are stimulated by an entrainment signal (zeitgeber) [13,17].

The objective of the current study was to examine the potential role of ID4 in the circadian system, specifically addressing its function in regulating photic entrainment of the clock. ID4 is structurally and functionally discrete from ID1, ID2, and ID3, exhibiting the highest structural divergence within the gene family [6,18]. Furthermore, unlike the other *Id* genes, *Id4* is expressed in a restricted body distribution pattern that includes the brain and SCN [6,7,8,9,10,11,12,18]. Not only is ID4 of interest in the context of the circadian system due to the functional role of ID2 having been established, but that *Id4*, along with *Id1*, *Id2*, and *Id3* mRNA, is expressed within the SCN in a 24-h rhythmic pattern and sharing a concordant peak phase [7]. Furthermore, *Id4* was recently identified as a highly expressed gene in a meta-analysis of gene enrichment within the SCN [19].

One aspect of endogenous circadian clocks is the requirement for minor adjustments on a daily basis to ensure their correct phase relationship with the environment, which is a process called entrainment. The daily alteration between light and dark is the major synchronizer, or zeitgeber, for the circadian clock in most organisms. The aim of our current studies was to explore whether ID4, similar to its paralog ID2, contributed to the process of circadian photoentrainment. This was undertaken using the *Id4*−/− mouse and by studying parametric (continuous) and nonparametric (discrete) models of photoentrainment [5,20,21,22]. Parametric entrainment is produced in response to light exposure of continuous durations and is based on a tonic response of the clock to the luminance level. This results in changes to the angular velocity of the clock and a change of the circadian period or cycle length [23,24]. In comparison, nonparametric entrainment is produced in response to light pulses of short duration and is based on a circadian-dependent response to light, which is also known as a phase response curve (PRC). This results in discrete changes in the phase of the clock [5,20,21,22].

In the current investigation, the locomotor activity rhythms of *Id4*−/− mice were examined under LD cycle and constant dark (DD) conditions, and mice were subjected to various photic entrainment challenges. At the molecular level the gene expression of *Id* genes within the SCN was quantified. We examined the anatomy of the SCN and retina including melanopsin-positive retinal ganglion cell (RGC) densities and the retinohypothalamic tract (RHT), as well as pupillary responses to light. Our results reveal an *Id4*-null mouse photoentrainment phenotype that likely occurs at the level of the SCN. The phenotype is not explained by major differences in the anatomy and function of the retina or retinal pathways or in changes to gene expression within the SCN of *Id2* or *Id3*. The characteristics of the phenotype are the opposite of that found for the *Id2*−/− mouse. Based on our prior studies of ID2 [7,13,17], we propose a related model in which ID4 acts on the clock in an opposing manner to ID2 to elevate its circadian photic response.

## 2. Results

### 2.1. Circadian Rhythm Characteristics Are Different in Id4−/− Mice

**Figure 1:**
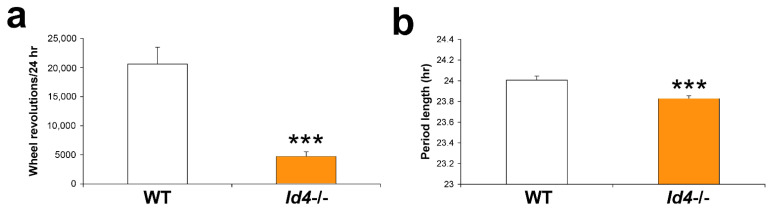
Mice null for the *Inhibitor of DNA binding 4* (*Id4*) gene (*Id4*−/− mice) show a reduction in the magnitude of wheel activity and a shorter free-running period length. (**a**) Wheel revolutions and (**b**) circadian period length for wild-type (WT) (white) and *Id4*−/− (orange) mice. See Figure 2, Figure 3 and Figure 4 for representative locomotor activity records (actograms). *** *p* < 0.001.

**Figure 2:**
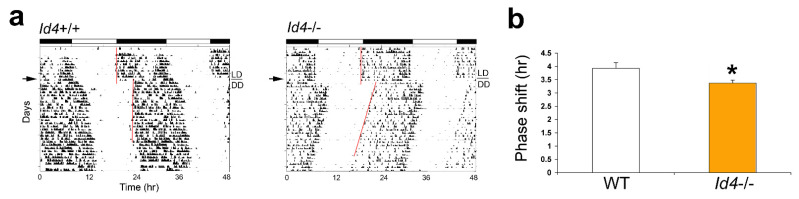
*Id4*−/− mice show a reduction in the magnitude of phase delays in response to a 10-h extension of the light phase. (**a**) Locomotor activity records of wild-type (*Id4*+/+) and *Id4*−/− mice exposed to a 10-h extension of the light phase of the light/dark (LD) cycle. Actograms are shown in double-plotted format with each horizontal line representing a 48-h period, and the second 24-h period plotted to the right and below the first. Vertical bars represent periods of wheel-running activity. Mice were exposed to 10 h of prolonged light exposure on day 1 and transferred to constant darkness (DD) for the remainder of the experiment. The line above DD on the right indicates the transition from LD to DD. The timing of the LD cycles is indicated by the white/black bars above the records. The arrow on the left indicates the actual day of treatment (Day 1). A line is fitted to the phase of activity onset for several days before and after the transfer to DD. (**b**) Mean ± SEM magnitude of the phase shifts produced by light treatment. Extrapolated activity onsets of the first day following the 10-h light treatment was used to determine the size of resultant phase delays. Wild type (WT; white), *n* = 19; *Id4*−/− (orange), *n* = 15. * *p* < 0.05.

**Figure 3:**
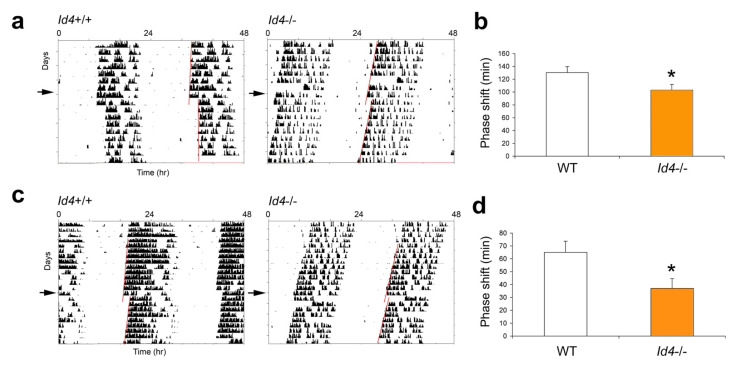
*Id4*−/− mice treated with a single SATURATING (30 min, 1000 lux) or a SUB-SATURATING (4 min, 8 lux) discrete light pulse at Circadian time 16 (CT16) exhibit a decrease in the magnitude of the phase shift. Wild-type (*Id4*+/+, WT) and *Id4*−/− mice (*n* = 20 and 16, respectively) were maintained in DD, and wheel-running activity was recorded. Mice were exposed to a saturating (**a**) or sub-saturating light treatment (**c**). The arrow on the left indicates the actual day of treatment. A line is fitted to the phase of activity onset before and after the light treatment, and the time difference between the two lines is the measured phase delay of the free-running rhythm. Mean ± standard error of the mean (SEM) magnitude of the phase shifts in wild-type (white) and *Id4*−/− mice (orange) produced by the (**b**) saturating and (**d**) sub-saturating light treatment (* *p* < 0.05).

**Figure 4:**
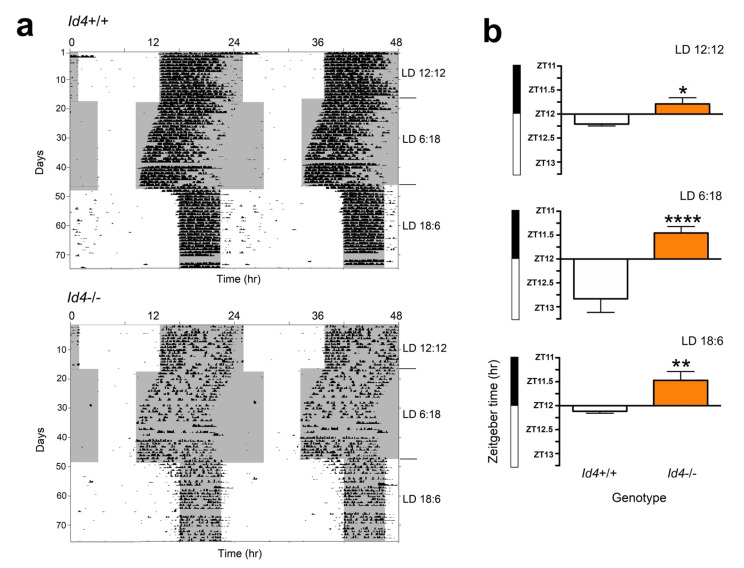
*Id4*−/− mice show an advance in the phase angle of activity onset relative to lights off tested under 12:12 LD, 6:18 LD, and 18:6 LD photoperiods. (**a**) Wheel-running locomotor activity records of wild-type and *Id4*−/− mice exposed to a 12:12 (standard) LD cycle for 1 month, transferred to 6:18 (short) LD cycle for another month, and finally exposed to an 18:6 (long) LD cycle for the remainder month of the experiment. The lines below each LD cycle on the right indicates the transition to each LD. The gray area indicates the dark period (scotophase) of each cycle. (**b**) Phase angle of activity onset to lights OFF (Zeitgeber time 12, ZT12) of wild-type (white) and *Id4*−/− mice (orange) on 12:12, 6:18, and 18:6 LD cycles. Values are group means ± SEM for wild-type (*n* = 20) and *Id4*−/− (*n* = 16) mice. Significant differences between genotypes were detected (* *p* < 0.05, ** *p* < 0.01, **** *p* < 0.0001).

*Id4*-mutant mice were tested for differences in the parameters of their circadian locomotor activity. Patterns of wheel-running activity were monitored in *Id4*−/− mice and wild-type littermate controls. All mutant mice expressed significant ≈ 24 h rhythms under LD cycle (daily or diel) conditions, and under constant dark (DD) (circadian free-running) conditions, as determined objectively by both Fourier and periodogram analyses. Under constant dark conditions, the *Id4*−/− mean free-running period length was found to be shorter by 11.5 min, with wild type of 24.0 h versus *Id4*−/− of 23.8 h (*p* < 0.0001; wild type, *n* = 18, *Id4*−/−, *n* = 13) (Figure 1). Mice also exhibited a marked reduction in their wheel-running activity as measured by mean wheel rotations/24 h, expressing a 75% reduction of the wheel revolutions compared to wild-type controls (*p* < 0.0001) (Figure 1). Unsurprisingly, the Fourier analysis *power spectrum* value, which equates to an objective measure of the relative strength of the rhythm/circadian amplitude, correlates with this activity level with a corresponding reduction (*p* = 0.0018; wild type, 0.2006 ± 0.04038, *n* = 18; and *Id4*−/−, 0.02833 ± 0.005279, *n* = 13; mean ± standard error of the mean (SEM)). In regard to other rhythm parameters, no difference was found between genotypes in the duration of the nocturnal bout of locomotor activity in each circadian cycle, also known as *alpha*, which was measured in DD following a 12:12 LD cycle, as an average of the multiple days of study (*p* = 0.3655, n.s.; wild type 12.52 ± 0.25 h versus *Id4*−/− 12.82 ± 0.22 h). Apart from a shorter circadian period length, locomotor rhythms in the mutant mice were found to be unremarkable. See Figure 2, Figure 3 and Figure 4 for representative locomotor activity profiles (actograms) of *Id4*−/− and wild-type mice monitored under LD cycle and DD conditions. Adult *Id4*−/− mice tended to have a lower body mass, as assessed sex-specifically in age-matched (4–6 months old) males (*p* = 0.0244; wild type, 37.9 ± 2.6 g, *n* = 6; *Id4*−/−, 30.2 ± 1.3 g, *n* = 6).

### 2.2. Id4−/− Mice Exhibit Reduced Phase Delays in Response to Continuous Light

To examine the effect of the *Id4*-null mutation on photic entrainment, we measured the phase shift entrainment response of mice to a large delay in the daily photoschedule. In our earlier *Id2*-mutant mice studies, when mice were exposed to 10 h of continuous light starting at Zeitgeber time 12 (ZT12) (time of lights off on a normal LD cycle) and then transferred into constant darkness to ascertain the magnitude of the resultant phase shift of the clock, *Id2*−/− mice exhibited an almost two-fold increase in the size of phase shifts relative to control mice [7,17]. In the current study, this specific LD to DD transition protocol was applied to *Id4*−/− mice. Mice were transferred to DD after a single long day representing the shift of the photoschedule. The magnitude of the shift produced by this single 10-h extension of the light phase was 3.9 ± 0.21 h in wild types and 3.37 ± 0.11 h in the mutant mice. Surprisingly, *Id4*−/− mice exhibited smaller phase delays (*p* = 0.0175), being 14% reduced in magnitude compared to wild-type controls (Figure 2).

### 2.3. Id4−/− Mice Show Smaller Phase Shifts in Response to Nonparametric Entrainment, Using Discrete Saturating Light Pulses and Discrete Low Illuminance/Short Duration Light Pulses

To further explore the response of the *Id4*−/− mice to the phase delay portion of the circadian cycle, we challenged mice under nonparametric entrainment conditions. In circadian theory, the effects of entraining agents (zeitgebers) have been modeled in two different ways. In continuous or parametric entrainment, the entraining agent acts continuously to adjust the oscillator period length so that rhythms in the exposed and unexposed organisms gradually move out of phase from one another. In contract, under discrete or nonparametric entrainment, the phase of the circadian oscillator is abruptly shifted to the new phase soon after exposure to the entraining agent. Based on these principles, the protocol of a 10-h continuous light extension of the photophase could have yielded shifts by either mechanism. However, the two models can be distinguished through the use of short discrete pulses of light. Therefore, to better characterize the role of ID4 in photoentrainment, mice were exposed to a discrete pulse of saturating light during early subjective night, which is a phase of the mouse phase response curve (PRC) showing predictably large phase delays [25]. Mice were maintained under constant darkness for 10 days before being treated with a 1000-lux 30-min light pulse at Circadian time (CT) 16 (or 4 h following the onset of free-running activity; with CT12 marking the activity onset). Then, mice were allowed to continue to free-run in DD (Figure 3). The magnitude of the shift produced by this single saturating light pulse was 130.4 ± 9.2 min in wild-type mice and 102.8 ± 9.0 min in the mutant mice. This treatment resulted in a 22% reduction in the magnitude of phase shifts in the *Id4*−/− mice compared to wild-type mice (*p* = 0.0254) (Figure 3a,b). Then, mice were tested at the same circadian phase for responses to a light pulse of low illuminance and short duration, specifically 8 lux for 4 min. This experiment also revealed differential responses in the *Id4*−/− mice. However, the magnitude of difference between genotypes was even greater (*p* = 0.0462), with the shift produced by this single low illuminance short duration light pulse being 65 ± 8.7 min in wild-type mice and 37 ± 7.6 min in the mutant mice. The 4 min 8 lux treatment in *Id4*−/− mice resulted in a 43% reduction in the magnitude of the phase shift compared to controls (Figure 3c,d).

### 2.4. Id4−/− Phase Angle Is Advanced under Variable Photoperiods

Previous work on the circadian system of *Id2*-null mice revealed differences in the phase angle of activity onset in the *Id2*−/− mice that was delayed relative to controls [7,14]. In the nonparametric model of entrainment, the phase angle of entrainment is the product of the daily phase shift, which is required for the circadian system to compensate for the difference between the endogenous period length (τ) and the period length of the zeitgeber (T), i.e., 24.0 h. According to the parametric model of entrainment, τ changes predictably with exposure to light. In organisms combining both models of entrainment, the phase angle of entrainment will depend on τ, which in turn will depend on the photoperiod length. We examined mice on different photoperiods, initially on a standard LD 12:12, then switching for 30 days to a short photoperiod of LD 6:18, and finally transitioning to a long photoperiod of LD 18:6 for 30 days (Figure 4). The phase angle of the activity rhythm was measured under the three different photoperiods, and it was consistently advanced in *Id4*−/− mice relative to the wild-type control group (two-factor ANOVA: effect of photoperiod F_2,72_ = 2.62, *p* = 0.0801, n.s.; effect of genotype, F_1,72_ = 47.60, *p* < 0.0001; interaction, F_2,72_ = 6.05, *p* = 0.0037) (Figure 4). Under the short photoperiod, the change in phase angle was especially large, being 83 min advanced relative to wild-type controls (*p* < 0.05) (Figure 4). The difference in the phase angle between the two genotypes was 25 min earlier for *Id4*−/− on 12:12 LD cycle, 83 min earlier under the short photoperiod, and 39 min earlier under the long photoperiod. The relationship between photoperiod and phase angle was different between the genotypes. While increasing daylength in wild-type mice correlated with an increased negative phase angle relative to lights off (ZT12.0) (one factor ANOVA, F_2,38_ = 7.074, *p* = 0.0024), in mutant mice there was no relationship between photoperiod and phase angle (ANOVA, F_2,34_ = 1.743, *p* = 0.1902, n.s.) (Supplementary Figure S1). In *Id4*−/− mice, the phase angle was consistently positive relative to lights-off and similar between daylengths.

### 2.5. Histological Analysis of Id4−/− SCN

**Figure 5:**
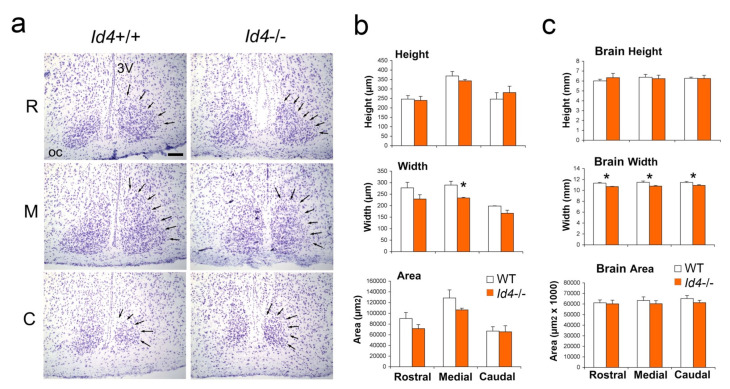
Histological analysis of suprachiasmatic nucleus (SCN) and whole brain shows anatomical differences between *Id4*−/− and wild-type mice. (**a**) Cresyl violet-stained coronal sections through rostral (R), medial (M), and caudal (C) aspects of *Id4*+/+ and *Id4*−/− SCN. Arrows indicate perimeter of the SCN. OC, optic chiasm; 3V, third ventricle. Scale bar = 100 µm. (**b**,**c**) Analysis of height, width, and area of rostral, medial, and causal aspects of SCN and whole forebrain, respectively. Brain assessed in coronal aspect. Values shown in the histogram are group means ± SEM for wild-type (WT) (white) and *Id4*−/− (orange) mice (wild type, *n* = 4; *Id4*−/−, *n* = 4). Significant differences were detected in width of SCN and forebrain between genotypes (* *p* < 0.05).

The hypothalamic SCN is the site of central circadian clock [1,2], and it is innervated by retinal inputs that regulate its photoentrainment. The developing and adult brain of the *Id4*−/− mouse has previously been reported to show certain deficits/defects, including enlarged ventricles and a generally smaller size [26,27]. Therefore, we examined the basic anatomy of the SCN by measuring its height (dorso-ventral plane), width (medial–lateral plane), and area of its boundaries as defined from the examination of nissel stained coronal sections taken through its length (Figure 5). The width in the medial region of the SCN was found to be smaller (*t*-test, *p* < 0.05). Consistent with this finding, when the dimensions of the entire *Id4*−/− brain were examined in the coronal plane, brain width, but not height or area, was found to be smaller in all sections assessed (*p* < 0.05). Due to the smaller width dimension in *Id4*−/− mice, cell counts were performed on coronal sections in the dorsomedial and ventrolateral (retino-recipient) SCN regions. While no differences were observed between genotypes in the dorsomedial SCN, cell density in the ventrolateral SCN was found to be lower in *Id4*−/− mice (*p* < 0.05) (Supplementary Figure S2). Otherwise, the basic histological architecture of the *Id4*−/− SCN was found to be unremarkable and similar to wild-type SCN.

### 2.6. Histological Analysis of Id4−/− Retinal Hypothalamic Tract

**Figure 6:**
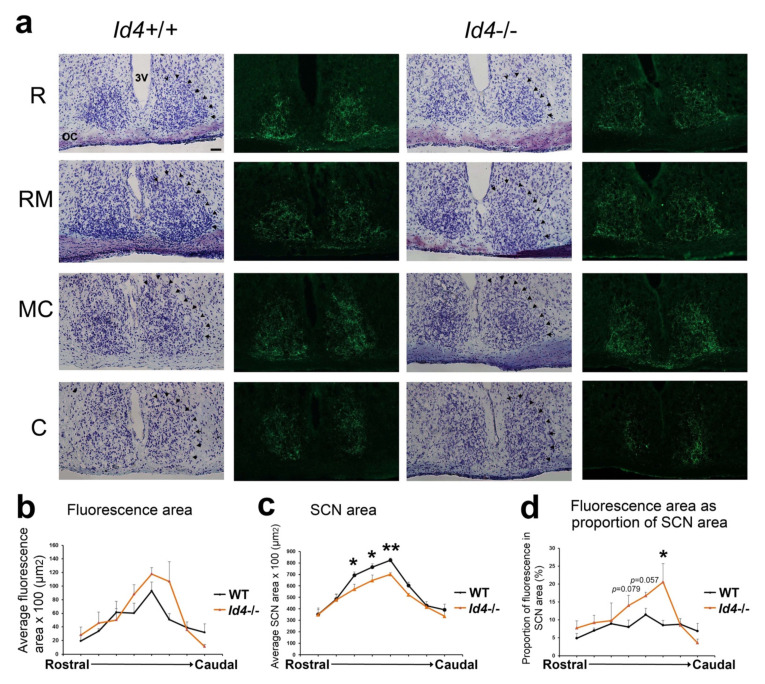
Tracing the retinohypothalamic tract (RHT) of *Id4*+/+ and *Id4*−/− mice. (**a**) Fluorescence and light microscopy images of the coronal aspect of the SCN from the rostral (R), through to rostral-medial (RM), medial-caudal (MC), and caudal (C) portions of the SCN. Arrows in cresyl violet-stained coronal sections indicate the perimeter of SCN. OC, optic chiasm; 3V, third ventricle. Scale bar = 50 µm. The terminal fields of the RHT are visible as green color (Alexa Fluor 488). The densely innervated central and ventro-lateral regions contain a preponderance of terminals from the contralateral retina. (**b**) Fluorescence (terminal field) through SCN (rostral to caudal), (**c**) SCN area, (**d**) fluorescence as a proportion of SCN. (wild type (WT; black), *n* = 5; *Id4*−/− (orange), *n* = 5). Significant differences were detected in fluorescence of the medial-caudal SCN and the area of the SCN between genotypes indicated by an asterisk (* *p* < 0.05, ** *p* < 0.01).

The SCN is innervated in its ventrolateral region by fibers of the retinal hypothalamic tract (RHT), which is a monosynaptic pathway originating from melanopsin-containing intrinsically photo-responsive retinal ganglion cells (ipRGCs). Due to the difference in SCN width and in ventrolateral regional cell density, we assessed the retinal innervation of the *Id4*−/− SCN using anterograde tract tracing using a fluorescent cholera toxin B subunit injected into the eye. Using this technique, we can observe the terminal field of the SCN retinal innervation (Figure 6a,b). Terminal distribution field of retinal input is not significantly different between genotypes (Figure 6). However, since the SCN is smaller in *Id4*−/− mice (Figure 6c) (see above and Figure 5), the proportion of SCN area containing retinal fibers is larger in the medial-caudal aspect of the SCN (Figure 6d).

### 2.7. Retinal Anatomy and Pupil Constriction Responses in Id4−/− Compared to WT Mice

**Figure 7:**
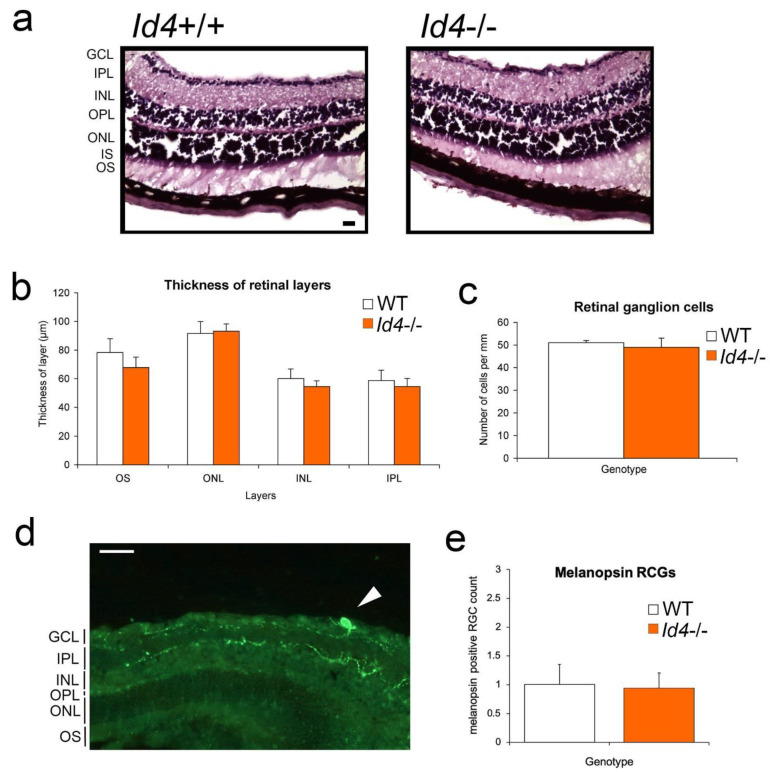
Retinal anatomy compared between *Id4*−/− and wild-type (*Id4*+/+, WT) mice. (**a**) Cresyl violet-stained 16 µm sections through representative wild-type and *Id4*−/− mouse retina showing layers. GCL, ganglion cell layer; IPL, inner plexiform layer; INL, inner nuclear layer; OPL, outer plexiform layer; ONL, outer nuclear layer; IS, inner segment; OS, outer segment layer. Scale bar = 20 µm. (**b**) Retinal layers were measured in µm. Mean ± SEM of retinal layers are shown, and differences between genotypes for each layer assessed by t-test (n.s.). (**c**) Number of retinal ganglion cells (RGCs) was counted per 1 mm length of retina. No significant difference was detected between genotypes in either the thickness of layers or in the number of RGCs (wild type (WT; white), *n* = 4; *Id4*−/− (orange), *n* = 6). (**d**) Melanopsin immunostaining in the retina. Representative fluorescent immunostaining of melanopsin cell soma (arrow) in the RGC layer of an *Id4*−/− mouse with neuron projection into the IPL. In addition, in this image are melanopsin-positive fibers present in the GCL and INL. Scale bar = 50 µm. (**e**) Number of melanopsin-positive RGCs (cell soma) was counted per 1 mm length of retina. Mean ± SEM number of cells is shown (wild type, *n* = 4; *Id4*−/−, *n* = 6), and no significant difference was detected between genotypes in the number of cells.

One possible explanation for the observed *Id4*−/− photoentrainment phenotype could be anatomical and/or functional changes to the retina and/or retinal hypothalamic tract (RHT), which are critical components of the SCN photic input pathway. Therefore, we examined this anatomy of the retina between genotypes by histological analysis. Qualitative review of the images and quantitative data analysis revealed no significant differences between the retinal layers of *Id4*+/+ and *Id4*−/− mice (*t*-tests, n.s.; wild type, *n* = 5, *Id4*−/−, *n* = 5; Figure 7a,b). Furthermore, there was no difference in the number of retinal ganglion cells (RGCs) between genotypes (n.s.) (Figure 7c), including when examined at the sub-regional level (left-lateral, right-lateral, and medial retina; data not shown). We also examined the melanopsin-positive intrinsically photoreceptive (ip) RGCs, since they represent an important contribution to accessory visual functions, namely circadian entrainment and pupillary response [21]. A representative image of fluorescent-immunostained melanopsin shows where ipRGCs are located in the retina and their low population density (Figure 7d), which is consistent with other reports [21]. No significant difference in the quantity of the ipRGCs, as counted by the number of cell soma, was detected between *Id4*+/+ and *Id4*−/− mice (n.s.) (Figure 7e), this being an average of ≈2% of the RGC population.

**Figure 8:**
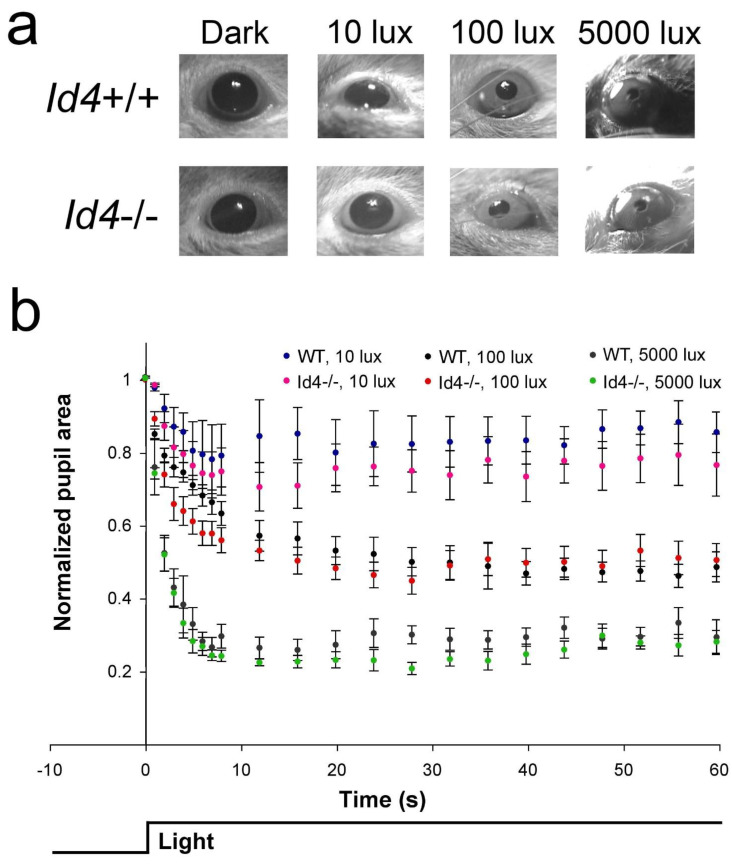
Pupil constriction responses are comparable between *Id4*−/− and wild-type (*Id4*+/+, WT) mice. Pupil constriction response to 60 s of white light. Animals were dark adapted for at least 2 h and treated between Zeitgeber time 8 (ZT8) and ZT11. (**a**) Representative animals after 24 s of light at 10, 100, and 5000 lux light intensities. The pupil/cornea ratio was normalized to the dark-adapted aperture from time zero (normalized pupil area = 1.0). (**b**) Mean ± SEM relative pupil diameter was assessed (wild type (WT), *n* = 6; *Id4*−/−, *n* = 7). Significance testing was performed by two-factor RM-ANOVA (n.s.). No differences were detected in the speed or magnitude of response at any of the three light intensities.

Projections of the ipRGCs and the RHT/retinothalamic tract are components of the retina and retinal pathway, respectively, and are shared by both the circadian and pupillary systems [21]. Therefore, to investigate the retina at the function level, we tested pupil constriction responses in dark-adapted *Id4*−/− mice at three different intensities of white light, 10, 100, and 5000 lux. Based the quantitative data of comparing relative pupil apertures over a period of 1 min (Figure 8a,b), there was no differences in either the rate or magnitude of pupil constriction in response to light between *Id4+/+* and *Id4−/−* mice at any light intensity (two-factor RM-ANOVAs: 10 lux, effect of time, F_21,231_ = 7.05, *p* < 0.0001; effect of genotype, F_1,11_ = 0.6460, *p* = 0.4386, n.s.; interaction, F_21,231_ = 0.5816, *p* = 0.9286, n.s.; 100 lux, effect of time, F_21,231_ = 88.23, *p* < 0.0001; effect of genotype, F_1,11_ = 0.4233, *p* = 0.5286, n.s.; interaction, F_21,231_ = 3.256, *p* < 0.0001; 5000 lux, effect of time, F_21,231_ = 126.0, *p* < 0.0001; effect of genotype, F_1,11_ = 2.088, *p* = 0.1763, n.s., interaction, F_21,231_ = 0.3676, *p* = 0.9955, n.s.). However, visual inspection of the data suggested an increased response at 3 to 8 s at 100 lux and an increased maximal response for the 5000-lux exposure. It is for this reason that the data for 4 s and at 28 s were examined specifically and compared at the three different light intensities. No significant differences between genotypes were observed (two-factor RM-ANOVAs: 4 s, effect of illuminance, F_2,22_ = 49.66, *p* < 0.0001; effect of genotype, F_1,11_ = 3.798, *p* = 0.0773, n.s.; interaction, F_2,22_ = 0.1963, *p* = 0.8232, n.s.; 28 s, effect of illuminance, F_2,22_ = 80.47, *p* < 0.0001; effect of genotype, F_1,11_ = 2.339, *p* = 0.1544, n.s.; interaction, F_2,22_ = 0.05507, *p* = 0.9465, n.s.) (Supplementary Figure S3).

### 2.8. Gene Expression of Id1 Is Abnormal in Id4−/− SCN

**Figure 9:**
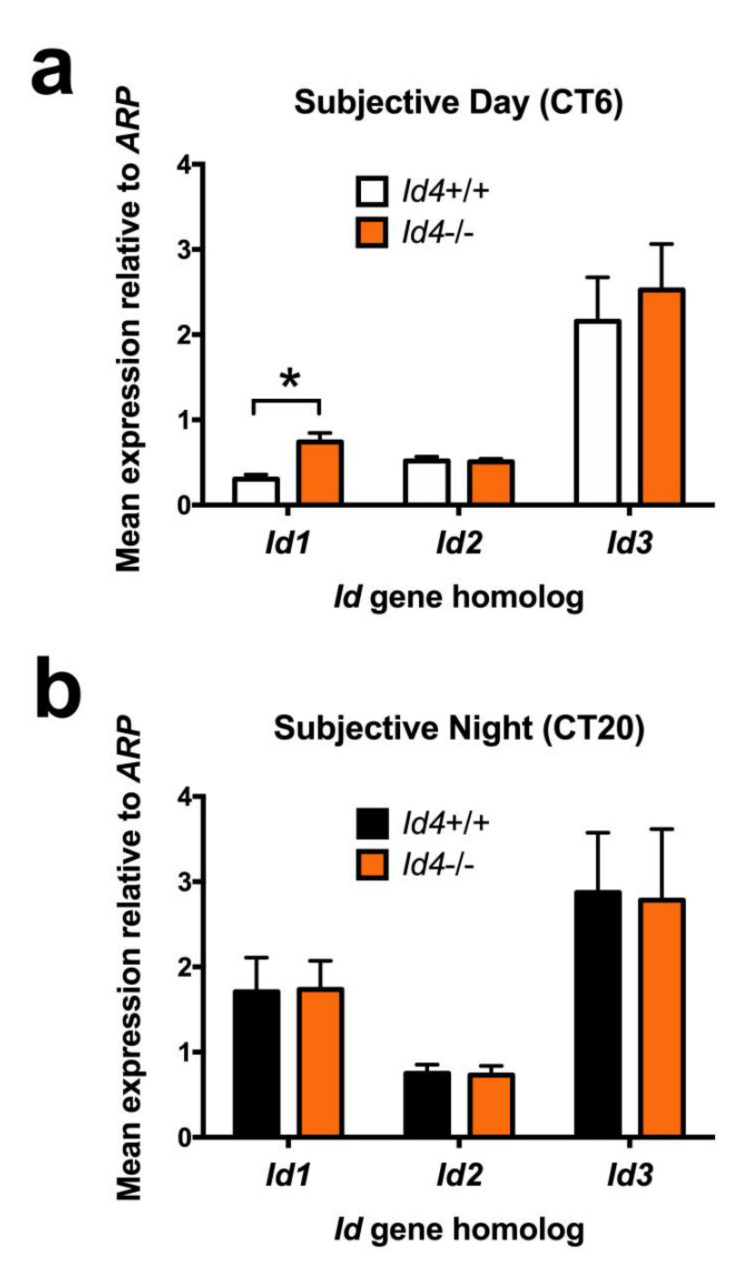
*Id4*-null mice exhibit abnormally elevated daytime gene expression of *Id1* within the suprachiasmatic nucleus. Quantative gene expression assessment of *Id1*, *Id2*, and *Id3* in wild-type and *Id4*−/− mice SCN. Mice were extrained to a 12:12 LD cycle and transferred to DD before time-specific assement. qRT-PCR analysis of time-specific SCN tissue punches, collected at (**a**) Circadian time (CT) 6 (subjective daytime, wild type (*Id4*+/+) *n* = 8, *Id4*−/− *n* = 7) and (**b**) CT20 (subjective night, wild type *n* = 10, *Id4*−/− *n* = 12). Values are mean ± SEM relative gene expression values (relative to *acidic ribosomal protein*, *ARP* expression). * *p* < 0.05.

All four *Id* genes are expressed in the SCN, are rhythmic, and share a similar peak phase [7]. We have established in in vitro studies that ID2, ID1, and ID3 can interact with canonical clock components CLOCK and BMAL1, as well as interfere with their transactivation potential. Furthermore, specifically demonstrated for ID2, this interaction is via the HLH domain, and it results in the sequestration of CLOCK and BMAL1 to the cytoplasm [7,13]. There is evidence from other systems that there is reciprocity between the *Id* gene homologs. Furthermore, in several biological systems, *Id4* has an opposing modulatory effect [6]. Therefore, one explanation for the *Id4*−/− photoentrainment phenotype is through an altered mRNA abundance of one of the other *Id* gene homologs. To test this possibility, punches of SCN tissue were extracted from mice under DD conditions at either CT6 (subjective day) or CT20 (subjective night), the respective predicted approximate nadir (CT4–CT8) and peak (CT16–CT20) of the *Id* gene rhythms [7]. Extracted RNA were subjected to real-time quantitative reverse transcription polymerase chain reaction (qRT-PCR) analysis. Consistent with previous studies, pairwise analysis revealed differences in expression based on time-of-day for *Id1* and *Id2* in wild-type mice, with higher expression values at CT20 versus CT6 (Student’s *t*-tests with Bonferroni MMC, *p* < 0.05). Similarly, *Id4*−/− mice expressed similar time-of-day differences (*p* < 0.05). No temporal differences were observed for *Id3* gene expression (n.s.). These results provided confidence in the SCN RNA extraction method to then evaluate potential genotypic differences. Genotypic comparisons revealed a two-fold elevation in *Id1* mRNA specifically at CT6, during the subjective daytime, and presumed *Id1* rhythm nadir (*p* < 0.05) (Figure 9a). No differences were observed between wild-type and *Id4*−/− mice in *Id2* or *Id3* mRNA abundance (n.s.) (Figure 9a,b).

## 3. Discussion

The molecular circadian clock is a key regulator of biochemistry, physiology, and behavior, adapting the body and coordinating its organ systems to routine changes over the 24-h day. Using a targeted deletion strategy, we have identified a previously unrecognized role for *Id4* in the circadian clock and its entrainment. The relevance of this finding is of significance since photoentrainment is the major mechanism by which the organism can shift its clock and synchronize to the external environment, and there are health implications when this process is compromised.

In this study, we have explored the photoentrainment phenotype of the *Id4*-null mouse, using protocols based on both parametric and nonparametric models of entrainment. We show distinct alterations in the entrainment properties of the clock in *Id4*−/− mice that are observed when the circadian system is probed with continuous light, discrete pulses of either saturating or sub-saturating light, which likely explain the advanced phase angle of entrainment observed under different photoperiods. *Id4*−/− mice also expressed a shorter free-running circadian period length and reduced locomotor wheel-running activity. Anatomical and functional (pupillometry) aspects of the retina and its pathways were examined and found to be normal in the *Id4*−/− mouse, suggesting that the observed differences in photoentrainment are not due to a developmental abnormality in the retina or RHT. Finally, using gene expression analysis of the *Id4*−/− SCN, we reveal a time-specific increase in *Id1* mRNA compared to wild-type counterparts. Together, our findings suggest that the reduced phase delays of the clock observed in *Id4*-null mice likely reflect decreased nonparametric entrainment responses [7,12,14,17].

Analysis of the photoentrainment response following exposure to a long continuous 10-h 250-lux exposure to light resulted in a 14% reduction in the magnitude of the resultant phase delay in mutant mice. *Id4*-null mice responded similarly to a discrete saturating light pulse (30 min at 1000 lux) delivered at CT16, and which is the presumed peak of the delay portion of the phase response curve [25]. This treatment resulted in a 21% attenuated phase shift response in the mutant mice. Then, we explored whether a light treatment of short duration and lower intensity might differentially impact *Id4*−/− mice. Similar to the response at CT16 using a pulse of long duration (30 min) and high illuminance (1000 lux), *Id4*−/− mice treated with a single sub-saturating light pulse (4 min at 8 lux) at the same circadian phase exhibited a dramatically smaller shift compared to wild types, being 43% attenuated. Consistently, all treatment regimens produced an attenuated phase shift of the circadian pacemaker.

Phase adjustments of the mammalian clock by light can be modulated by factors such as the length of exposure, and the normal circadian system undergoes a change in its state of photosensitivity or responsiveness over the course of a photic stimulus. The relationship between duration of the light pulse and the magnitude of resultant phase shift follows a linear/log trajectory during the first hour after light onset. However, after one hour, it follows a linear/linear relationship [28,29,30]. Therefore, the circadian system is initially very responsive to light exposure, especially 0 to 15 min, but after 1 h, the response becomes weaker. Despite the change, the system remains responsive for the duration of a long exposure, e.g., 10 h. It is during exposure to the extremes of photic stimuli that wild-type animals are known to be in these different physiological states of responsiveness. Therefore, it is surprising that the reduced phase responses of the *Id4*−/− mice are revealed when challenged by a short duration low intensity (4 min, 8 lux), intermediate duration high intensity (30 min, 1000 lux), or long duration treatment (10 h, 250 lux). In contrast to these findings in *Id4*-null mice, the *Id2*−/− circadian system expresses a phase response phenotype only when challenged with extremes of light exposure (dim short exposure versus bright long exposure). The most striking difference between the *Id* gene mutants is the *Id2*−/− phase response is an *increase* in the magnitude rather than a *decrease* as found in *Id4*−/− mice. Another important independent variable in contributing to the phenotype is irradiance [28,30], and separating the factors ‘duration’ and ‘intensity’ will be important in further investigations.

Analysis of the phase relationship of the *Id4*−/− locomotor activity onset under three different photoperiods revealed that the phase angle was advanced relative to wild-type controls in LD 12:12, long, and short photoperiods, and occurring earlier than lights off (ZT12.0). Despite changes in the length of the photophase, in *Id4*−/− mice, there was no corresponding change in the phase angle. This was different from the response of the wild-type mice, which showed an increasingly delayed phase angle the shorter the photophase. Under short days, the difference between *Id4*−/− and wild-type mice was as much as 83 min. The difference in phase angle between wild-type and *Id4*−/− mice could be explained by a different parametric response to light in the different genotypes; i.e., different photoperiods could induce different changes in period length (tau) [5,22,31,32]. In other animal and human studies, an increased deviation of endogenous period length away from the environmental T-cycle (24-h LD cycle) or a decreased strength of the zeitgeber (light intensity) often results in marked increases in the phase angle [5,22,31,32]. A popular simple rule is that a short period length produces an advanced phase relationship with the cycling environment, although there are exceptions to this predictive/correlative relationship [33]. In this study, we revealed that *Id4*−/− mice expressed a shorter free-running period length. However, it is unclear whether the 11.5 min shorter period length expressed by *Id4*−/− mice would entirely explain the observed advanced phase angle results: The size of the phase angle differences observed between genotypes is large and does not change in *Id4*−/− mice as the photophase increases.

These findings of *Id4*−/− photoentrainment are particularly interesting since *Id2*−/− mice express an opposite phenotype: *Id2*-null mice express a delayed phase angle compared to wild-type mice, and the *Id2*−/− phase angle difference cannot be explained by a change in tau, as the free-running period length is similar to that of their wild-type controls [7,12,14,17]. A possible explanation that would be consistent with the findings of the current study and the findings in *Id2*−/− mice is that the fixed light intensity used in the experiments effectively has a reduced zeitgeber strength in the *Id4*-null circadian system, thereby increasing the phase angle and advancing it relative to wild-type controls. This phenotype is also exaggerated when we compare the phase angle of *Id4*−/− and wild-type mice on a short LD 6:18 photoperiod. Therefore, it is reasonable to consider that the different phases of entrainment can be explained by weaker phase-delaying effects in *Id4*−/− mice, which would move the early subjective night toward the evening light.

Other noteworthy features of the behavioral rhythms of the *Id4*-null mice are a reduction in wheel-running activity as measured by wheel revolutions per unit time; and unsurprisingly, the mice also express reductions in the *power* measure of rhythmicity assessed by Fourier analysis. Interestingly, these findings are similar to those described for *Id2*−/− mice [7,14]. While not systematically measured throughout the current study, *Id4*−/− mice tended to be smaller, which is a finding consistent with a related study [34]. In the current study, a subgroup analysis of *Id4*−/− adults revealed a ≈20% smaller body mass compared to wild-type counterparts. Fat depot analysis of *Id4*-null mice suggests that a major component of the reduced body mass phenotype is specifically in this body compartment [34]. A reduced body weight and abnormal adipose tissue storage are also observed in *Id2*−/− mice, which are also significantly smaller than wild-type controls [7,9,14,15,16]. Therefore, a difference in body mass or disturbance to energy metabolism might contribute, at least in part, to the reduced quantity of *Id4*−/− wheel-running locomotor activity.

In an earlier study focused of the role of *Id2* in the circadian system, it was established that ID2 contributed to the circadian photoentrainment mechanism [7]. A major phenotype detected in this study was a large ≈80% increase in the magnitude of a phase shift in response to a long 10-h continuous exposure of light at night, as well as an increased speed of entrainment when exposed to a phase-delaying change in time zones. As part of this investigation, a cohort of *Id4*-null mice were subjected to locomotor activity analysis under LD 12:12 and constant dark conditions and then assessed for phase shift responses to this 10-h continuous light challenge. No significant differences were detected in period length in DD or in the magnitude of the phase shifts in response to the light treatment. These findings are inconsistent with the current study, and it was originally concluded that there was an absence of a photoentrainment phenotype [7]. However, in light of the current study, this conclusion was clearly a premature determination. Careful examination of the prior work reveals a smaller mean average in the phase shift response (wild type, 3.79 ± 0.18 h, *n* = 11; heterozygote, 4.20 ± 0.24 h, *n* = 9; and *Id4*−/− 3.50 ± 0.09 h, *n* = 7); in fact, there was a 12% lower mean in *Id4*−/− versus wild-type and heterozygote mice. This finding, while below the level of statistical significance, is consistent with the current results.

In this first and limited analysis of circadian behavior in *Id4*-null mice [7], experimentation was conducted on a smaller cohort of mice, and this included an additional third group (heterozygotes). In the current study, the results of the 10-h extended light protocol and the 11.5 min shorter period length determinations are still subtle responses. There was a tendency for a smaller phase shift response in the original study [7], but this did not reach statistical significance. The purpose of inclusion of the *Id4*−/− mice in this earlier study was to compare with *Id2*−/− mice, which exhibited a large change in phase shift magnitude and in the opposite direction (increase) [7,17]. In light of this background, and using a larger cohort of mice, a circadian phenotype is observed: In the current study, *Id4*-null mice of a larger sample size were exposed to three different photic entrainment challenges, and a consistent effect of the *Id4* genotype was detected in response to all treatment paradigms (long continuous light of intermediate illuminance; brief exposure to dim light; and intermediate duration exposure with high illuminance). Note that in the earlier study, *Id4*−/− mice were only challenged with the 10-h light exposure [7]. In the current study, this protocol results in the smallest difference observed between genotypes (14% or 32 min). In comparison, the other two photic challenges resulted in larger 21% and 43% magnitude changes. Not to dispel the significance to circadian function (these are substantive effects on circadian rhythmicity), the differences measured between *Id4* genotypes in these two parameters (period length and phase shift in response to a 10-h light treatment) are of relatively small magnitude, which makes the task of elucidating differences a challenge. This is in comparison with some of the larger period length and phase shift responses observed in mice bearing mutations for particular canonical clock genes, e.g., *cry1*, *cry2*, *per1*, and *per2* single knockout strains [35,36,37,38]. Coupled with the related results of the phase angle/photoperiod experiments, the authors are confident that there is a consistent and distinct circadian photoentrainment phenotype expressed in the *Id4*-null mouse.

It is possible that these findings reflect changes in development, although several pieces of evidence suggest that ID4 contributes to a post-mitotic function in the adult circadian system (see below). One possibility is that the photoentrainment phenotype is due to a developmental aberration occurring at the level of the SCN, such as in its cell proliferation, differentiation, and/or cellular localization [6]. Importantly, histological analysis of SCN coronal sections showed no gross anatomical difference between *Id4*−/− and wild-type littermates, indicating that any circadian phenotypes in the mutants are not due to a gross developmental defect in the basic organization of the SCN. The reduced medial-lateral (width) dimension in the SCN when examined in the coronal plane, and reduced cell density within the ventrolateral retinorecipient region, highlight the possibility of an abnormal regional organization. However, since the entire forebrain is also smaller in this specific dimension, the finding in the SCN dimension is not unique to this nucleus but a generalized feature of the entire *Id4*−/− forebrain. Further work would have to be conducted to explore the possibility of an abnormal regional organization.

Pupillometry and retinal structure analyses indicate that the *Id4*-null mouse photic system is similar in its sensitivity or responsiveness compared with wild-type counterparts. These results of retinal anatomy are similar to that observed for the *Id2*-null mouse; i.e., there was no difference between mutant and wild-type control mice [17]. Additionally, no differences were observed in the pupil responses of *Id2*−/− mice when challenged by the same light intensities (10 and 100 lux) and assessed by pupillometry [17]. Despite the normal gross anatomy and pupil constriction responses, it is possible that a difference in the *Id4*−/− circadian system still might occur at the retinal level [29,39]. As *Id4*−/− pupillary responses are normal, it is unlikely that an abnormal retinal function underpins the muted phase shift responses to short-duration/low-intensity photic stimuli. However, it is possible that an altered *Id4*−/− retinal process, such as adaptation, contributes to generating the abnormal circadian responses observed with photic exposure of greater duration and illuminance. If a factor, it likely involves a complex contribution from different classes of photoreceptor cells, namely rods, cones, and ipRGCs, and their signal integration [40,41]. An important area of future work will be to further address any retinal contribution to the phenotype or to determine that the anatomical locus of the phenotype resides entirely within the SCN.

Tract-tracing analysis of the *Id4*−/− RHT revealed a similar density of terminal fibers innervating the SCN region as wild-type controls and in the typical and well-described ventrolateral retinorecipient region. As noted above, histological analysis of SCN revealed a smaller *Id4*−/− SCN in the medial-lateral (width) dimension when examined in the coronal plane. However, this finding is consistent with the finding of a reduced forebrain size in the same dimension; and a smaller brain with enlarged ventricles has been reported previously in the *Id4*−/− mouse [26,27]. Due to this anatomical feature, the *Id4*−/− RHT afferents appear to innervate a disproportionally larger region of the SCN, and it is plausible that this may impact features of the circadian system such as the photoentrainment response.

To assess whether the absence of ID4 in the mutant mice results in an altered pattern of expression of the other members of the *Id* gene family, we quantified gene expression in the *Id4*−/− SCN at the presumed peak and nadir phases of their endogenous rhythm [7]. *Id1* mRNA was found to be elevated specifically during the subjective daytime and nadir phase of its circadian cycle. Our observations suggest a mechanism by which ID4 regulates the expression of *Id1*, which in turn can interact with CLOCK and BMAL1 [13], thereby interfering with the normal operation of the clock, and in particular their contribution to the photic induction of *period* gene expression and subsequent execution of phase shifts [17]. As ID1 can interact with CLOCK and BMAL1, these data suggest the possibility for an indirect effect of ID4 upon the circadian clock via changes in levels of ID1. Lines of evidence from other biological systems would support this cooperative regulatory mechanism model in which *Id* gene products influence the expression of *Id* gene paralogues [6,18]. For example, in chick ovary granulosa, ectopic overexpression of ID2 decreases *Id1*, *Id3*, and *Id4* mRNA, while the blockage of *Id2* expression is associated with increased *Id1*, *Id3*, and *Id4* mRNA [42]. In *Id4*−/− mice prostate, *Id1* mRNA is elevated, although in normal prostate, *Id4* is highly expressed and contrasts with weak or absent expression of *Id1* and *Id3* [43]. There is an inverse association between ID1 and ID4 in tumorigenesis [6]. Clearly, the interrelationships and transcriptional regulatory networks between *Id* genes are complex. For example, there are overlapping functions of ID4 and ID2, which both bind the bHLH proteins retinoblastoma, OLIG1, and OLIG2 [6,18].

In the previous investigations on the role of *Id2* and *Id4* in photoentrainment, the induction of all four *Id* genes in the SCN by qRT-PCR was examined after an acute light treatment delivered during the early subjective night [7]. The positive control immediate early genes *c-fos* and *mPer2* were induced, peaking at 30 and 75 min, respectively. However, no increase or decrease in expression levels were observed for any *Id* gene at these time points, including *Id4*. This suggests that an acute induction or suppression of *Id4* mRNA is not part of the mechanism responsible for the observed *Id4* photoentrainment phenotype.

In our earlier investigations of protein interaction and transcriptional activation assays between ID proteins and canonical clock components, unlike for ID1, ID2, and ID3, we were unable to provide evidence of an interaction between ID4 and CLOCK or ID4 and BMAL1 [7,13]. This suggested a unique feature for ID4 in its potential action in the molecular circadian clock. Another possible mode of action for ID4 is through its inhibition of other ID paralogues through direct binding, i.e., acting as an inhibitor of a transcriptional inhibitor. Evidence for this to occur has been revealed in cancer cell lines [44]. ID4 can heterodimerize with ID1, ID2, or ID3 via its HLH domain, allowing neutralization of their dominant negative regulation of bHLH transcription factor activity. If this were to occur in the molecular clock, the consequences would be regulation of ID-CLOCK and ID-BMAL1 interactions, thereby influencing the overall quantity of available CLOCK–BMAL1 heterodimers [7,13,17]. This might impact the negative limb TTFL of the clock, including *period* genes and their inducibility by photic stimuli, as well downstream output components of the clock that regulate the rhythmicity of clock-controlled genes [7,13,17,45,46]. In this model, the *Id4*-null mutation would be predicted to increase free ID1, ID2 and ID3, thereby decreasing CLOCK:BMAL1 heterodimer abundance and its nuclear accumulation [7,13]. This would be predicted to result in a decreased photoentrainment response, and it might also influence core clock function, such as produce an altered circadian period length. This is what is observed in the current study with *Id4*−/− mice expressing a shorter period length.

Much of our understanding of ID regulation of the circadian system comes from studies focused primarily on ID2. These investigations have revealed a contribution of ID2 in circadian regulation at the three fundamental levels of the clock, namely its input (including entrainment), core clock function, and output [7,9,12,13,14,17]. A proposed mechanism of action is through direct binding, sequestration to the cytoplasm, and inhibition of the bHLH factors CLOCK and BMAL1 [13,17]. In the context of photoentrainment specifically, *Id2*−/− mice exhibit rapid photoentrainment responses, exaggerated phase shift responses, a delayed phase angle of entrainment, as well as increased photic-induced *per1* gene expression within the SCN [7,17]. Consistent with these findings in the intact animal, serum-stimulated *Id2*−/− fibroblasts in vitro show higher levels of induced *per1* expression [13]. As *per1* is a light-inducible state variable of the clock, its inducibility is directly associated with the magnitude of phase shifts, and it is induced/suppressed by various zeitgeber signals [1,2]. It is in this context that we began to investigate the potential contribution of ID4 to the circadian system. In the SCN, *Id4* is rhythmically expressed and phase concordant with the three other members of the *Id* gene family [7]. Based on a genome-wide meta-analysis of enriched gene expression in the adult SCN versus whole brain, *Id4* was identified as one of the most abundant genes [19]. This perhaps highlights its key contribution specifically in SCN function.

## 4. Materials and Methods

### 4.1. Animals

*Id4*−/− (*Id4*-null) mice were generated and maintained, including PCR genotyping, as described previously [7,14,26]. Mice were generated from in-house breeding at the University of Notre Dame (UND) and maintained on their C57BL/6J background. *Id4*−/− mice were generated from heterozygote x heterozygote crosses, and age and sex-matched littermate *Id4*+/+ (wild-type, WT) mice were used as controls in all procedures. Food and water were available ad libitum. Unless otherwise indicated, mice were housed in a 12-h light/12-h dark (LD) regimen under climate-controlled conditions (19–21 °C, 50–65% humidity). All mice were entrained to the LD cycle for at least 3 weeks prior to experimentation. Experiments were conducted in accordance with the IACUC at UND. The *Id4*-null mouse line was donated to the Mutant Mouse Resource & Research Centers (MMRRC) and is available from the Jackson Laboratory (Bar Harbor, ME) (41569-JAX; *Id4*^tm1Mais^/Mmjax).

### 4.2. Locomotor Activity Monitoring, Behavioral Manipulations, and Circadian Phenotype Analysis

Adult mice (≥3 months of age) were maintained in individual cages (29 × 11.5 × 13 cm) equipped with a running wheel (Actimetrics, Wilmette, IL, USA). Mice were studied under a 12:12 LD cycle (150–400 lux, fluorescent lights: General Electric 36-W cool white), with lights on at 0700 h and off at 1900 h, or in constant darkness (DD) (0 lux). Mice were maintained on a 12:12 LD cycle for at least 10 days to establish stable entrainment and then transferred to DD for 30 days to measure free-running rhythms. Wheel-running activity was monitored by a PC computer and using *Clocklab* hardware and software (Actimetrics). Fourier (Fast Fourier Transformation) and X^2^ periodogram analyses were conducted on activity data using the *Clocklab* software to test for the presence of a ≈24-h rhythm in DD. Actogram analyses were conducted as described previously [7,17]. Briefly, the activity data were processed with the *Clocklab* software and with data arranged into 6 min time bins. Lights off was defined as Zeitgeber time (ZT) 12. Circadian time (CT) 12 indicated activity onset under continuous darkness, which was determined using standard methods [7,47]. Briefly, estimation of CT12 was determined by drawing a line by eye through at least 7–10 consecutive activity onsets on double-plotted actograms. To ensure for consistency, period length under free-running conditions was determined using both the periodogram analysis and determined from the slope of this fitted line. All lines were fitted by two individuals who were independent of the experimenter and who were blind to the treatments. The first 3–5 days in DD were removed from analysis to allow for stabilization of rhythms following transfer from LD to DD. *Activity level* was measured as wheel revolution counts/24 h, averaged across 10 continuous days. Fourier analysis generates a *power spectrum* value, which equates to an objective measure of the strength of the rhythm/circadian amplitude, was generated from a 10-day segment in DD. The duration of the nocturnal bout of locomotor activity in each circadian cycle, also known as *alpha*, was measured in DD and calculated as an average of 10 days.

### 4.3. Continuous/Parametric Entrainment Experiments

In the *phase delay* experiment, the LD cycle was extended by 10 h so that lights off changed from 1900 h to 0500 h. The protocol was followed as previously described [7,17]. The light intensity during the extension of the light phase was 150–250 lux, matching that of the preceding LD cycle. The magnitude of the phase delays was determined from actograms by drawing a line of best fit through 7–10 days of activity onsets immediately before the light treatment and a second line through 7–10 days after the treatment. The phase shift was calculated as the difference between the actual onset of activity and that predicted by the pre-treatment line on the last day of treatment.

### 4.4. Discrete/Nonparametric Entrainment Experiments

In the acute light pulse experiments, mice were entrained to a 12:12 LD cycle for 14 days and transferred to DD. The protocol was followed as previously described [7,17]. Briefly, after 14 days in DD, mice were exposed to a saturating pulse of white light (30 min at 1000 lux), starting at CT16, and maintained for at least a further 10 days in DD. Then, the mice were re-entrained to an LD cycle for at least 14 days, transferred to DD for 14 days, and then challenged with a light pulse of lower intensity and duration (4 min at 8 lux) also starting at CT16, and maintained for at least a further 10 days in DD. The magnitude of the phase delays was determined as described above in Section 4.3. Light intensities were established using neutral density filters (Lee Filters, Burbank, CA, USA).

### 4.5. Phase angle Determination under Different Photoperiods

Mice were maintained on the photoperiods LD cycle 12:12 (normal), LD 6:18 (short days), and LD 18:6 (long days), each for 30 days and in sequence. Using actograms, the phase angle of activity onset relative to ZT12 was calculated from the last 10 days of continuous activity in LD in each respective photoperiod and by drawing a line of best fit through 7–10 days of activity onsets.

### 4.6. Whole Brain and SCN Histology

Animals were sacrificed by cervical dislocation, and brains were frozen on dry ice and stored in a –80 °C freezer prior to sectioning on a cryostat. Coronal sections (16 μm) were collected on adhesive-coated slides (Fisher Scientific, Pittsburgh, PA, USA) followed by a 4% para-formaldehyde treatment for 5 min and two 1xPBS washes. Slides were rehydrated in a descending series of alcohols (3 min each of 100%, 95%, and 70%), briefly dipped in ddH_2_O, and then immersed in Cresyl Violet (Sigma-Aldrich, St. Louis, MO, USA) for 10–20 min. Sections were dehydrated in an ascending series of alcohols (30 sec in each of 70%, 95%, and 100%), cleared in xylene, and finally coverslipped with DepexR (VWR International, West Chester, PA, USA). Cresyl Violet (Nissl) stained sections were studied under brightfield microscopy and images captured digitally (Diagnostic Instruments, Sterling Heights, MI, USA). Three representative sections per animal were analyzed through the bilateral SCN region, representing rostral, medial, and causal zones. Height (dorsal-ventral dimension) and width (medial-lateral dimension) (μm) and area (in μm^2^) of left and right SCN were measured using the SPOT software program (Diagnostic Instruments), and the mean of these values was calculated.

### 4.7. Retinal Hypothalamic Tract (RHT) Tracing

A tract-tracing procedure was adapted from Duffield et al. (1995) [48], using a fluorescent-labeled Cholera Toxin Subunit B conjugate. Mice were anaesthetized with tribromoethanol/tertiary amyl alcohol (10 µL/g body weight, i.p.). The right eyeball was gently protruded, and a small incision was made through the sclera with a 25-gauge hypodermic needle. A 10 µL Hamilton syringe was introduced to allow 5 µL of a 10 mg/mL solution of cholera toxin B subunit conjugated to Alexa Fluor 488 (Molecular Probes, Thermo Fisher Scientific) in PBS to be injected into the vitreous humor. After recovery, mice were returned to the controlled photoperiod rooms and were perfused 5 days later. Mice were deeply anaesthetized with tribromoethanol (15 µL/g body weight, i.p.), given 500 units of heparin intracardially, and perfused through the ascending aorta with PBS for 3 min followed by 4% paraformaldehyde for 5 min. Brains were dissected out, placed in 4% paraformaldehyde overnight, and then cryopreserved in 20% sucrose in PBS overnight. Brains were cut in the coronal plane on a cryostat at 16 µm. Sections were washed in PBS, mounted on gelatin-coated glass slides, and imaged immediately using a florescent microscope. Immunofluorescence images were collected on a Leica DM500B microscope and digitally captured (Diagnostic Instruments). Alternate sections were stained by Cresyl Violet and prepared as described above (Section 4.6). These sections were dried, dehydrated in 70%, 95%, and 100% ethanol, and delipified with xylene; then, slides were coverslipped with DepexR. The sections were imaged by brightfield microscopy and digitally captured. Analysis of images was conducted using Image J software (NIH) to calculate dimensions (in μm^2^) of the SCN and its retinal afferents.

### 4.8. Retinal Histology and Anti-Melanopsin Immunohistochemistry

Retinal histology and anti-melanopsin immunohistochemical analysis of the retina were conducted as previously described [17,49]. Briefly, slides were prepared as described in Section 4.6 above, except coronal sections (16 μM) were collected and processed. Nissl-stained sections were viewed under brightfield microscopy and images were digitally captured. Retinal layers were measured in µm and RCGs counted using *SPOT software* (Diagnostic Instruments, Sterling Heights, MI, USA). Melanopsin immunostaining in the retina was performed on alternate 18 µm retinal sections, and immunofluorescence images were collected on a Leica DM500B microscope. Retinal ganglion cells and anti-melanopsin RGCs were counted.

### 4.9. Pupillometry

Pupil constriction response to 60 s of white light was assessed using methods outlined previously [17,50,51]. Briefly, mice were subjected to one minute of light at ZT8.5-11 to test the response and magnitude of pupil constriction. Mice were dark-adapted for at least 2 h before the test. Treatments consisted of 10, 100, and 5000 lux of white light. The mice pupil responses were recorded on a handheld video camera and analyzed by measuring pupil diameter in relation to cornea diameter. The pupil/cornea ratio was normalized to the dark-adapted aperture from time 0 s to provide a *measurement of relative pupil aperture* (normalized pupil area = 1.0).

### 4.10. SCN Gene Expression Analysis

SCN tissue was extracted as previously described [7]. Briefly, animals were transferred from a 12:12 LD cycle to DD for one day and sacrificed by cervical dislocation at CT6 and CT20; then, brains were rapidly removed and snap frozen in isopentane on dry ice before a 1 mm coronal section was cut at the level of the optic chiasm using a twin blade cutter. An SCN punch from a frozen slice was taken under a stereomicroscope using a flat-tipped 25G needle (internal diameter ≈0.5 mm) and tissue stored on dry ice. The shape of the optic chiasm and third ventricle were used to define the SCN region. RNA extraction was performed as described previously [4,7,8]. Briefly, SCN tissue was homogenized with Trizol reagent (Invitrogen, Carlsbad, CA, USA) following the manufacturer’s protocol. RNA quality was assessed by nanodrop RNA integrity analysis; qRT-PCR analysis was performed to quantify gene expression of *Id1*, *Id2*, and *Id3* using primer pairs [7]. RNA was DNaseI treated (Invitrogen), and cDNA was synthesized using a Taqman Reverse Transcriptase kit (Applied Biosystems, Foster City, CA, USA) and primed with random hexamers. PCR thermocycling and qRT-PCR were performed as previously described [4,7] using SYBR green reagent and an ABI PRISM 7500 Sequence Detection System, with quantification based on the generation of standard curves. Dissociation curves using the using Dissociation Curve software (ABI) were generated to test for primer dimers. Normalization of gene expression was calculated relative to acidic ribosomal phosphoprotein (ARP) [4,7].

### 4.11. Statistical Analysis

Statistical analysis was performed using GraphPad Prism software (GraphPad Software Inc., San Diego, CA, USA). Data are presented as mean ± SEM. Differences were analyzed by Student’s t-test or by one-way ANOVA, two-way ANOVA, or two-factor repeated measures (RM) ANOVA, followed by Tukey’s post-hoc test for multiple comparisons. *p*-value < 0.05 was considered statistically significant.

## 5. Conclusions

In this study, *Id4*-null mice exhibit a photoentrainment phenotype, which is characterized by reduced phase delays to discrete light pulses and to continuous light, and an advanced phase angle of activity onset relative to the time of lights off. These phenotypic differences are the opposite to those observed in *Id2*−/− mice. *Id4*−/− mice also exhibit a shorter period length and reduced wheel running locomotor activity. No gross changes in the retina were observed in *Id4*−/− mice nor were pupil constriction responses found to be different. Consistent with gross morphological changes in brain size, the SCN was found to be significantly smaller in *Id4*−/− mice. Retinal innervation of SCN of *Id4*−/− is similar to wild-type mice, although the receptive field of the RHT may be larger, since the SCN is proportionally smaller in *Id4*−/− mice. *Id1* expression is elevated in *Id4*−/− SCN. As ID1 can interact with CLOCK and BMAL1, these data suggest the possibility for an indirect effect of ID4 upon the circadian clock via changes in levels of ID1. It is plausible that this elevation in *Id1* mRNA and/or the absence of ID4 might result in changes in interactions with the bHLH canonical clock components or with targets upstream and/or downstream of the clock. These results reaffirm that *Inhibitor of DNA binding* genes play an important role within the circadian system and complement previous findings focused primarily on the *Id2* gene.

## Data Availability

The data presented in this study are available upon request from the corresponding author.

## Supplementary Materials

The following are available online at https://www.mdpi.com/article/10.3390/ijms22179632/s1, Figure S1: The relationship between photoperiod and phase angle of activity onset is different between the genotypes, Figure S2: Cell count analysis of the SCN of wild-type and *Id4*−/− mice, Figure S3: Pupil constriction responses of wild-type and *Id4*−/− mice analyzed at 4 s and 28 s after onset of light treatment.
