# Supplementary material for: Targeted Disruption of the Inhibitor of DNA Binding 4 (Id4) Gene Alters Photic Entrainment of the Circadian Clock
Source: Int J Mol Sci. 2021 Sep 6;22(17):9632. doi: 10.3390/ijms22179632 (PMC8431790; doi:10.3390/ijms22179632)
Supplement: Supplementary file 1 [file ijms-22-09632-s001.zip › Figure S1.pdf]

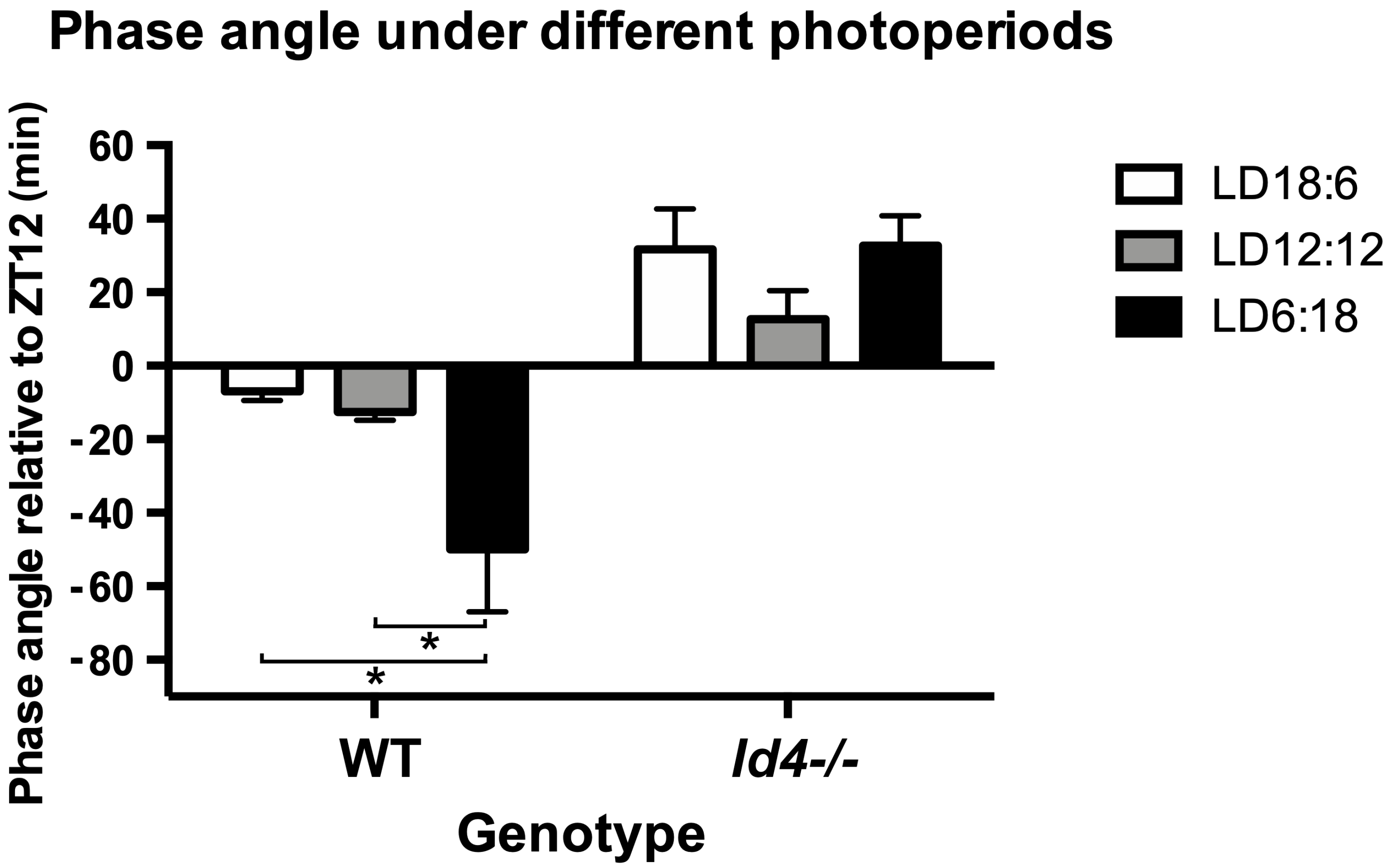

Figure S1. The relationship between photoperiod and phase angle of activity onset is different between the genotypes. Phase-angle of activity onset relative to lights OFF (ZT12) of wild-type (WT) (left) and *Id4*-/- (right) mice on 18:6, 12:12, and 6:18 LD cycles. Values are group means ± SEM for wild-type (n = 20) and *Id4*-/- (n = 16) mice. Increasing daylength in wild-type mice correlated with an increased negative phase angle relative to lights off (ZT12.0) (*$p < 0.05$). No differences between photoperiods were detected for *Id4*-/- mice, which consistently expressed a positive phase angle (n.s.). See Figure 4 for representative actograms and pairwise genotypic comparisons of wild-type and *Id4*-/- mice.
