# Supplementary material for: Targeted Disruption of the Inhibitor of DNA Binding 4 (Id4) Gene Alters Photic Entrainment of the Circadian Clock
Source: Int J Mol Sci. 2021 Sep 6;22(17):9632. doi: 10.3390/ijms22179632 (PMC8431790; doi:10.3390/ijms22179632)
Supplement: Supplementary file 1 [file ijms-22-09632-s001.zip › Figure S2.pdf]

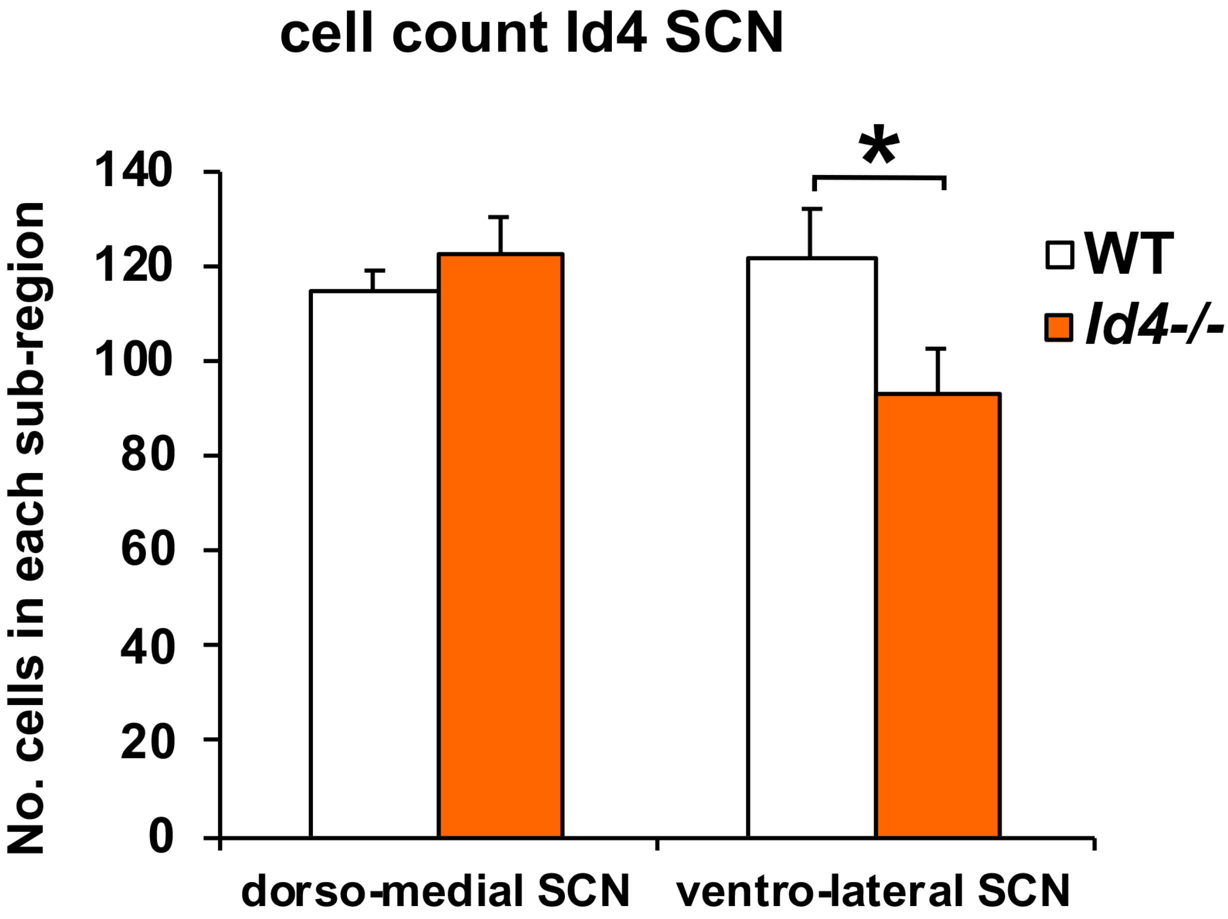

Figure S2. Cell count analysis of the suprachiasmatic nucleus (SCN) of wild-type and *Id4*-/- mice. Cells count analysis was performed on cresyl violet stained coronal sections in the dorsomedial and ventrolateral (retino-recipient) SCN regions using SCN-subregion templates. Values are group means ± SEM for wild-type (WT) (white) and *Id4*-/- (orange) mice (wild type, $n = 4$; *Id4*-/-, $n = 4$). Significant differences were detected in cell density of SCN between genotypes in the ventrolateral region (*$p < 0.05$). See Figure 5 for representative histological coronal sections through the SCN region of wild-type and *Id4*-/- mice.
