# Supplementary material for: Targeted Disruption of the Inhibitor of DNA Binding 4 (Id4) Gene Alters Photic Entrainment of the Circadian Clock
Source: Int J Mol Sci. 2021 Sep 6;22(17):9632. doi: 10.3390/ijms22179632 (PMC8431790; doi:10.3390/ijms22179632)
Supplement: Supplementary file 1 [file ijms-22-09632-s001.zip › Figure S3.pdf]

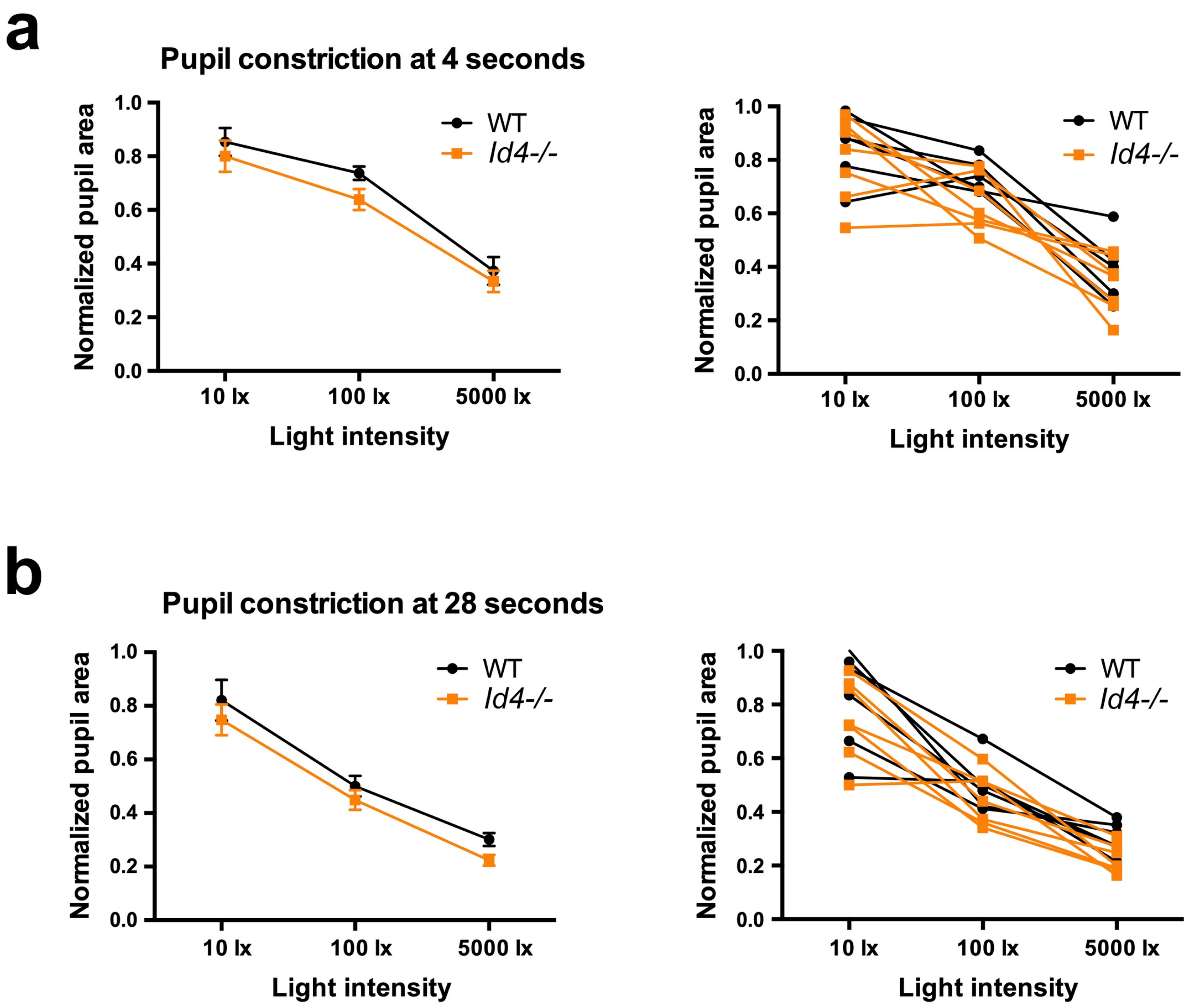

Figure S3. Pupil constriction responses of *Id4-/-* and wild-type mice analyzed at 4 seconds and 28 seconds after onset of light treatment. The response data at 4 and 28 seconds was examined specifically and compared at the three different light intensities (10, 100 and 5000 lx). Values are mean ± SEM relative pupil diameter (wild type (WT), n = 6; *Id4*-/-, n = 7). No significant differences between genotypes were observed (Two-factor RM-ANOVAs at 4 sec and at 28 sec). See Figure 8 for wild-type and *Id4*-/- mice representative pupil constriction responses and pairwise genotypic comparisons at all time intervals.
